# Heat Stress Regulates the Expression of Genes at Transcriptional and Post-Transcriptional Levels, Revealed by RNA-seq in *Brachypodium distachyon*

**DOI:** 10.3389/fpls.2016.02067

**Published:** 2017-01-10

**Authors:** Shoukun Chen, Haifeng Li

**Affiliations:** ^1^State Key Laboratory of Crop Stress Biology for Arid Areas, College of Agronomy, Northwest A&F UniversityYangling, China; ^2^Xinjiang Agricultural Vocational Technical CollegeChangji, China

**Keywords:** RNA-seq, heat stress, DEGs, *Brachypodium distachyon*, alternative splicing

## Abstract

Heat stress greatly affects plant growth/development and influences the output of crops. With the increased occurrence of extreme high temperature, the negative influence on cereal products from heat stress becomes severer and severer. It is urgent to reveal the molecular mechanism in response to heat stress in plants. In this research, we used RNA-seq technology to identify differentially expressed genes (DEGs) in leaves of seedlings, leaves and inflorescences at heading stage of *Brachypodium distachyon*, one model plant of grasses. Results showed many genes in responding to heat stress. Of them, the expression level of 656 DEGs were altered in three groups of samples treated with high temperature. Gene ontology (GO) analysis showed that the highly enriched DEGs were responsible for heat stress and protein folding. According to KEGG pathway analysis, the DEGs were related mainly to photosynthesis-antenna proteins, the endoplasmic reticulum, and the spliceosome. Additionally, the expression level of 454 transcription factors belonging to 49 gene families was altered, as well as 1,973 splicing events occurred after treatment with high temperature. This research lays a foundation for characterizing the molecular mechanism of heat stress response and identifying key genes for those responses in plants. These findings also clearly show that heat stress regulates the expression of genes not only at transcriptional level, but also at post-transcriptional level.

## Introduction

The natural environment for plants includes a set of abiotic and biotic stresses, and the responses to these stresses are complex (Cramer et al., 2011). Among the common abiotic stresses, high temperature stress is a major factor that influences plant growth and development (Rodriguez et al., 2015). Heat stress induces a series of physiological changes, such as an increased degree of lipid peroxidation, which decreases the thermal stability of the cell membrane (Mishkind et al., 2009; Narayanan et al., 2016); Blum et al. (2001) reported that the yield of wheat (*Triticum aestivum*) was positively correlated with the thermal stability of the cell membrane. Heat stress also produces a large number of free-radicals in chloroplasts and mitochondria, which can damage the membrane system of cells (Guan et al., 2013; Song et al., 2014). Additionally, the activity of radical-scavenging enzymes, such as superoxide dismutase (SOD), peroxidase (POD), and catalase (CAT) decreases and the content of malondialdehyde (MDA) increase. As a result of the release of free radicals, cells damage and even apoptosis can occur (Bita and Gerats, 2013; Hasanuzzaman et al., 2013).

The growth and development of some plant organs are affected by these responses to heat stress. For example, heat stress influences the development of inflorescences (Suzuki et al., 2014) and floral organs in cereal crops (Wu et al., 2015), especially the development of the male reproductive organs and the viability and germination of pollen grains (Young et al., 2004). The yield of these crop plants also is affected. A crop simulation model demonstrated that a variation of 2°C in average growing-season temperatures could lead to up to 50% yield reductions in grain production in wheat (Asseng et al., 2011). Additionally, during the filling stage in wheat, high temperature can influence the accumulation of organic matter and starch (Stratonovitch and Semenov, 2015). In general, high temperature could lead to catastrophic losses in cereal crops (Bita and Gerats, 2013).

*Brachypodium distachyon* is an important model plant for studying grasses (Draper et al., 2001), as it has a fully sequenced genome (International Brachypodium Initiative, 2010). It is an specially important model because the *B. distachyon* genome is close to wheat and its relatives (Subburaj et al., 2015). Previous studies have shown that high temperature results in several aspects in *B. distachyon*. First, it influences the growth and reduces the tiller numbers. Second, it disrupts the development of anthers and pollen (Harsant et al., 2013); high temperature had an effect on anther length and dehiscence, as well as the development of pollen. Additionally, results show that with the increase in daytime temperature, the number of pollen grains decreases, and when the temperature reaches 36°C, most pollen development is arrested at the uninucleate stage. In addition to the effect on pollen development, pollen viability, and deposition, the germination on the stigma is also affected by high temperature (Harsant et al., 2013). As a result, the setting rate and the harvest index of *B. distachyon* decreased under heat stress.

With the increased occurrence of extreme heat events, the effects on plant growth and development are becoming a severe issue for agriculture. It is imperative to identify the molecular mechanism that drives the response to heat stress. Previous studies have shown that the plant response to high temperature is associated with multiple processes and mechanisms involving heat shock proteins (HSPs), transcription factors (TFs), and other stress related genes (Qin et al., 2008; Liu et al., 2012; Hasanuzzaman et al., 2013), as well as changes in protein domains, phylogenetic relationships, and gene and protein structures (Cao J. et al., 2016). Recent studies in *B. distachyon* found that some genes are regulated by heat, such as 14-3-3 gene family (Cao H. et al., 2016), PP2C gene family (Cao J. et al., 2016), TIFY family genes (Zhang et al., 2015), NAC gene family (You et al., 2015) and bZIP TFs (Liu and Chu, 2015). High-throughput sequencing is an accessible and widely used technology that allows the sequencing of an entire transcriptome or genome in a timely and cost-effective manner (Reon and Dutta, 2016). Transcriptome studies of heat stress effects on rice, wheat, tomato, and grape have been reported (Frank et al., 2009; Liu et al., 2012; Kumar et al., 2015a; Wu et al., 2015). Studies in rice found that heat responsive genes in rice panicles (Zhang et al., 2012) and flag leaves (Zhang et al., 2013) were mainly involved in transcriptional regulation, transport, protein binding, and antioxidant and stress responses. Frank et al. (2009) found that HSPs, reactive oxygen species (ROS) scavengers, hormones, and sugars were involved in responses to heat stress in maturing tomato (*Solanum lycopersicum*) microspores. Liu et al. (2012) reported that the number of heat stress-regulated genes was almost twice the number of recovery-regulated genes, and that these heat stress-related transcriptional changes regulate a large number of important traits and biological pathways in grape (*Vitis vinifera* L.). In wheat, the combination of heat and drought stress acted in a synergistic manner rather than an additive manner (Liu et al., 2015). The regulatory network analysis of heat shock transcription factors (HSFs) and dehydration responsive element binding (DREBs) suggest that they were involved in the regulation of drought stress, heat stress, and combined heat-drought stress responses.

Since high temperature resulted in abnormal development of anthers, pollen, and setting rate, and harvest index, we were interested in investigating key genes responsible for resistance to heat stress. We used RNA-Seq to characterize differentially expressed genes (DEGs) not only in leaves of seedlings, but also in leaves and inflorescences of *B. distachyon* at the heading stage. We found that the heat stress not only regulates the expression level of genes, but also regulates alternative splicing (AS) of genes. Our findings lay a foundation for understanding the molecular mechanisms involved in response to heat stress.

## Materials and Methods

### Plant Materials, Growth Conditions, and Treatments

*Brachypodium distachyon* Bd-21 was grown in an artificial climate chamber at 26/22°C (day/night) with a photoperiod of 16/8 h (day/night). Two-week-old seedlings and eight-week-old plants at the heading stage were selected for heat stress treatment that was applied after plants were transferred to a growth chamber at 42°C. Seedling leaves were collected at 0 h (CK1) and 5 h (S1), heading-stage leaves were collected at 0 h (CK2) and 1 h (S2), and inflorescences were collected at 0 h (CK3) and 1 h (S3) after heat treatment, respectively. After collection, samples were frozen immediately in the liquid nitrogen and stored at -80°C until further use. Every sample had two biological replicates that were sequenced independently.

### RNA Extraction, Libraries Construction, Transcriptome Sequencing and Mapping

Total RNA was extracted using a TRIZOL kit (Sangon Biotech, Shanghai, China) according to the manufacturer’s protocol and treated with DNaseI (Sangon Biotech, Shanghai, China). Sequencing libraries were prepared with the Ion Total RNA-Seq Kit v2 according to the protocol (Life technologies, USA). Emulsion PCR was performed using the cDNA library as a template. The Template Positive Ion PI^TM^ Ion Sphere^TM^ Particles were enriched and loaded on the Ion PI^TM^ chip for sequencing. In total, 12 samples were sequenced.

The online bioinformatics program, FAST-QC^1^, was used to evaluate the quality of the sequencing data. After quality testing, clean reads were mapped to the reference genome of *B. distachyon* with Mapsplice software (Wang K. et al., 2010), in which the core Bowtie program identified the exon–exon splicing accurately.

### Identification of Alternative Splicing

After mapping, an ‘accepted_hits.bam’ file was generated that included information regarding the chromosome position for exonic reads and exon–exon junction reads. All junction reads had to meet two criteria: (i) a read must match perfectly to each of the two flanking regions of a potential junction site with more than 6-nt; and (ii) a junction site that has >3 non-redundant reads in both the CK and S samples must be filtered. Afterward, an ASD (Alternative Splicing detector) (Zhou et al., 2014) was developed to fulfill the following tasks: (a) reconstruction of exon-clusters based on the aforementioned reannotated transcriptome for identifying modes of AS events for each exon-cluster; (b) count the number of junction reads that align either to the inclusion or exclusion isoforms in both the CK and S samples, and calculate the *P*-value using junction read-counts between CK and S samples by the Fisher exact test; (c) calculate read coverage for the alternative exons and their corresponding genes in both CK and S samples, and calculate a second *P*-value by the Fisher exact test based on the alternative exon read coverage relative to the gene read coverage between CK and S samples; and (d) combine the two *P*-values to get an adjusted *P*-value using a weighted arithmetic equation for assessing the statistical difference in AS between CK and S sample groups.

### Identification of DEGs

With a log-fold expression change of log2FC > 1 or <-1, and a threshold of false discovery rates (FDR < 0.05), DEGs were filtered using a DEGSeq algorithm (Wang L. et al., 2010).

### GO Analysis

Gene ontology (GO) was applied to analyze the main functions of DEGs according to the GO database and to determine the biological implications of unique genes in the significant or representative profiles (Ashburner et al., 2000). The GO annotations from NCBI^2^, UniProt^3^, and Gene Ontology^4^ were downloaded Fisher’s exaction and χ^2^ tests were used to classify the GO categories with a FDR (Dupuy et al., 2007) calculated to correct the *P*-value. The smaller the FDR, the smaller the error there was in judging the *P*-value.

### Pathway Analysis

The identification of significant pathways for DEGs was according to the Kyoto Encyclopedia of Genes and Genomes (KEGG) database^5^. A Fisher exaction test was used to screen the significant enrichment pathways with a threshold of significance defined by *P*-value and FDR. The resulting *P*-values were adjusted using the FDR algorithm. Then, pathway categories with FDRs < 0.05 were reported. Enrichment provides a measurement of the significance of the genes function: as the enrichment increases, the corresponding function is more specific. This helped us identify more significant pathways from our DEG results.

### Identification of TFs

The *B. distachyon* TF database PlantTFDB 3.0^6^ (Jin et al., 2014), was used as a reference TF database. Putative TFs in *B. distachyon* were identified using BLASTx with a cut-off *E*-value of 1 × 10^-5^ (Kalra et al., 2013).

### Validation of Gene Expression

To validate the RNA-Seq data, the expression of selected up- or down-regulated genes, and alternative splicing events were confirmed by quantitative reverse transcription–polymerase chain reaction (qRT-PCR) or semi-quantitative RT-PCR analysis. qRT-PCR reactions were carried out as described previously (Liu et al., 2016) and every reaction was performed in triplicate. Data acquisition and analysis were performed using the QuantStudio^TM^ Real-Time PCR Software (ThermoFisher Scientific). Samples were normalized using *ACTIN* gene expression (Hong et al., 2008) and relative expression levels were determined using the 2(-ΔΔCt) analysis method (Livak and Schmittgen, 2001).

For semi-quantitative RT-PCR analysis, the concentration of template was first normalized using *ACTIN* expression (Hong et al., 2008). Subsequently, 15-μl reactions containing 7.5 μl 2 × Taq MasterMix (ComWin Biotech, Beijing), 0.75 μl of each forward and reverse primer, 0.5 μl cDNA (5.0 ng/μl), and 5.5 μl ddH2O. PCR cycling conditions began with 94°C for 5 min, and were followed by 40 cycles of 94°C for 30 s, 58-64°C for 30 s, 72°C for 30 s, and 72°C for 10 min.

## Results

### Quality of RNA-seq Data

To characterize DEGs responsive to heat stress in *B. distachyon*, we performed RNA-Seq on twelve samples using the Ion Proton^TM^ System. For each transcriptome, a total of 13.4–17.1 million clean reads were collected. Since the size of the *B. distachyon* genome is about 272-MB, and the poly(A)-tail mRNA size should be about 2.6–5.2-MB (1–2% of genome size). In the current study, the data from each *B. distachyon* sample were more than 2-GB, which could cover the poly(A)-tail mRNA for 384 to 770 times. Previously, 6,399 genes were reported to be involved in abiotic stress (Priest et al., 2014). Among these genes, 6,320, 6,320 and 6,330 genes were detected in our S1, S2, and S3 samples, respective. These results indicate that our data were sufficient for further analysis. Evaluation of the data quality showed that the length and the GC content of the reads were in accordance with the criteria (ReadsFilter > 90%, GC > 48%) (Supplementary Table S1). Additionally, more than 93% of the filtered reads mapped to the annotated reference genome (Supplementary Table S1). The raw data were submitted to the NCBI database under the SRA accession number: SRS1792875.

### Identification and Validation of DEGs

**FIGURE 1:**
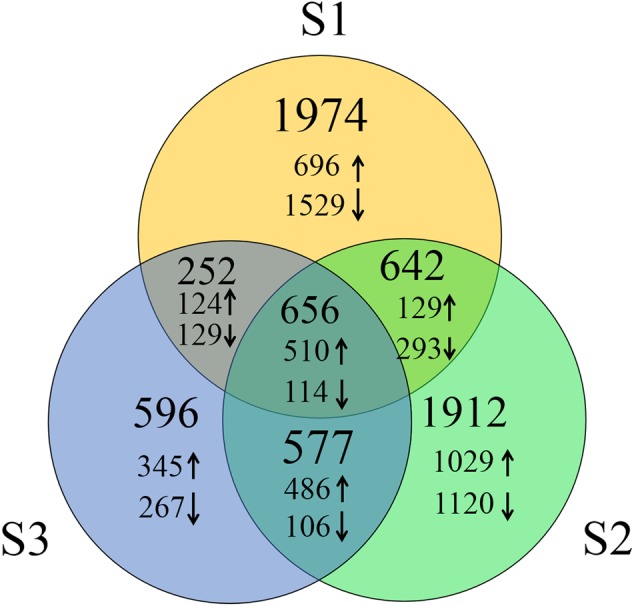
**Number of DEGs of three groups of heat treated samples.** Up arrows and down arrows indicate the overlap up-regulated and down-regulated genes respectively.

**FIGURE 2:**
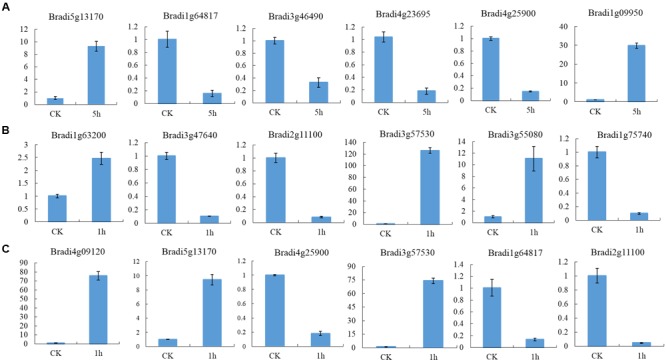
**Expression profile of randomly selected DEGs. (A)** Relative expression level of six genes in leaves of seedlings, **(B)** Relative expression level of six genes in leaves and **(C)** inflorescences at heading-stage, respectively.

To identify DEGs, we used the RPKM method (Reads per KB per Million reads) to calculate the expression levels of the unigenes. There were 3,524 (1,459 up-regulated, 2,065 down-regulated), 3,787 (2,154 up-regulated, 1,633 down-regulated) and 2,081 (1,465 up-regulated, 616 down-regulated) DEGs characterized in our three groups, respectively. In total, 6,609 DEGs were identified and of them, the expression of 656 (510 up-regulated genes and 114 down-regulated) genes were altered in all three groups of sample groups simultaneously (**Figure 1**). To verify the expression level of the DEGs, RT-qPCR was conducted, and the expression level of 13 randomly selected genes was analyzed. Results show that the expression trends were consistent with the RNA-Seq data (**Figure 2**, Supplementary Table S2).

Although only a few *B. distachyon* genes were reported to function in heat stress resistance, several genes from wheat, rice, and maize are thought to be responsible for resistance to heat stress. Therefore, we focused our analysis on the expression of their homologues in *B. distachyon.*

In rice, *DREB2B* encodes a TF that when it was overexpressed in *Arabidopsis*, the transgenic plants display an enhanced resistance to heat stress (Matsukura et al., 2010). In our sequencing data, the expression level of the homolog in *B. distachyon* (*Bradi2g29960*) for the rice *DREB2B* and maize *ZmDREB2A* genes was up-regulated in S1, S2, and S3. The expression of *TaMBF1c*, which encodes a multiprotein bridging factor, is induced by heat stress and confers heat tolerance in transgenic rice (Qin et al., 2015). The expression of *BdMBF1c* was up-regulated 80.211, 2751.3, and 358.86757 times in S1, S2, and S3, compared with control, respectively, Another wheat gene, *TaHsfA6f*, was reported to activate the expression of its downstream genes that are responsible for heat tolerance (Xue et al., 2015). In our data, the expression of the *TaHsfA6f* homologue in *B. distachyon* was elevated by 1.2476, 11.2309, and 99.564263 times in S1, S2, and S3, respectively, compared with that in the control. These results indicated that the identified DEGs included genes responsible for responding to heat stress.

### Hierarchical Clustering Analysis and Functional Enrichment Analysis of the DEGs

**FIGURE 3:**
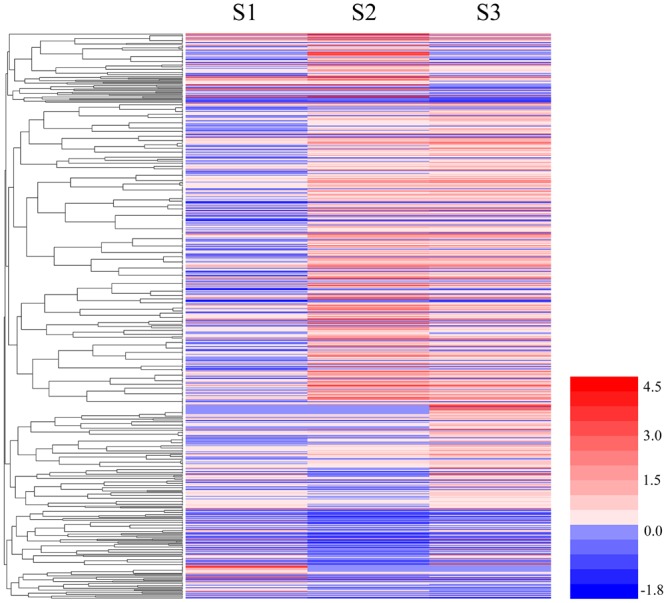
**Cluster analyses of DEGs among S1, S2, and S3.** The color key represents the value of log10 (RPKM). Red represents highly expressed (up-regulated) genes, while blue represents down-regulated genes.

Genes with similar patterns of expression can often have overlapping biological functions, and these similar genes may share functionality or participate in the same biological processes (BP; Huang et al., 2015). We used the RPKM expression values to analyze gene expression and perform a hierarchical clustering analysis of the gene expression patterns in 656 DEGs using HemI 1.0 (Deng et al., 2014) and the results are shown in **Figure 3**.

**FIGURE 4:**
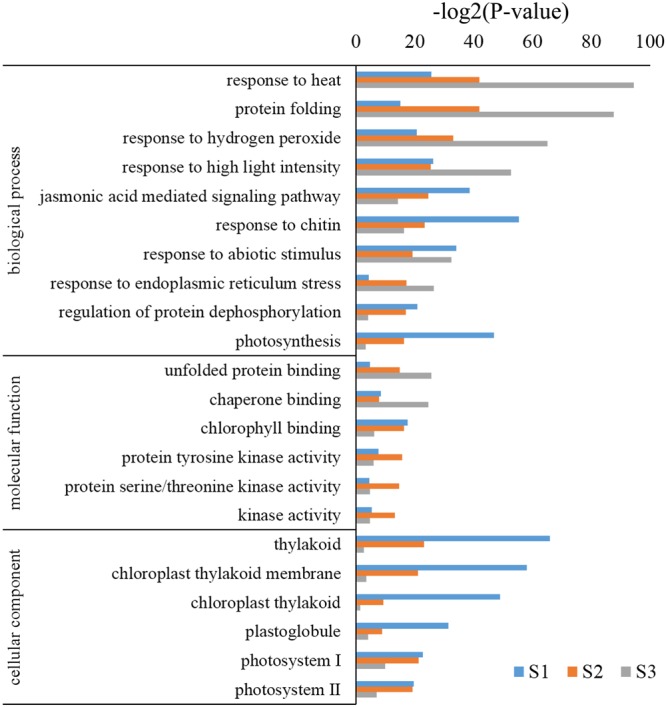
**Analysis of GO enrichment for DEGs**.

Gene ontology assignments were used to predict the functions of *B. distachyon* unigenes by classifying them by various BPs. Genes were classified into categories with three independent ontologies including biological process, molecular function (MF), and cellular components (Ashburner et al., 2000). Based on the results of the unigene annotation and GO analysis, 3,221, 3,389, and 1,890 unigenes were annotated to 1,763, 2,699, and 1,392 significant biological process GO terms (*p* < 0.05). Among these BPs, genes highly enriched in S2 and S3 were GO:0009408 (response to heat), GO:0006457 (protein folding), GO:0042542 (response to hydrogen peroxide), and GO:0009644 (response to high light intensity); while GO:0010200 (response to chitin) and GO:0015979 (photosynthesis) were highly enriched in S1 (**Figure 4**). Moreover, the cellular components category consisted of 330, 329, and 227 GO terms (*p* < 0.05) in S1, S2, and S3, respectively. Among them, GO:0009579 (thylakoid), GO:0009535 (chloroplast thylakoid membrane), GO:0009522 (photosystem I), and GO:0009523 (photosystem II) were highly enriched in S1 and S2, but not in S3 (**Figure 4**). MFs consisting of 1,149, 1,027, and 798 GO terms (*p* < 0.05) and highly enriched terms included GO:0016168 (chlorophyll binding) in S1 and S2, and GO:0051087 (chaperone binding) and GO:0051082 (unfolded protein binding) in S3 (**Figure 4**). These results suggest that different types of genes respond to heat stress at different developmental stages in *B. distachyon.*

KEGG is a database of biological systems and resource that integrate genomic, chemical, and systemic functional information (Kanehisa et al., 2012). All DEGs were screened in the KEGG database for pathway annotation and the significance level was calculated using the Fisher’s exact test (*p* < 0.05). In total, 23.60% (760/3,221), 20.51% (695/3,389) and 21.11% (399/1,890) of the differentially expressed unigenes in S1, S2, and S3 samples were annotated, respectively. There were 31, 18, and 16 pathway categories found to be significantly enriched in each developmental stage, as well. As shown in Supplementary Table S3, in S1 and S2, the most significantly enriched genes were antenna proteins (PATH:00196) involved in the photosynthetic pathway. In S3, the most significantly enriched genes were involved in protein processing pathways in the endoplasmic reticulum (PATH:04141). Additionally, genes encoding components of the spliceosome (PATH:03040) were expressed in S1 and S3 at high levels, and genes associated with photosynthesis, including PATH:00196 (Photosynthesis-antenna proteins), PATH:01200 (Carbon metabolism), PATH:00710 (Carbon fixation in photosynthetic organisms) and PATH:00195 (Photosynthesis) were also highly expressed. These results are consistent with the fact that leaves are the main organs to conduct photosynthetic reactions.

### Identification and Verification of Differentially Expressed Transcription Factors

**FIGURE 5:**
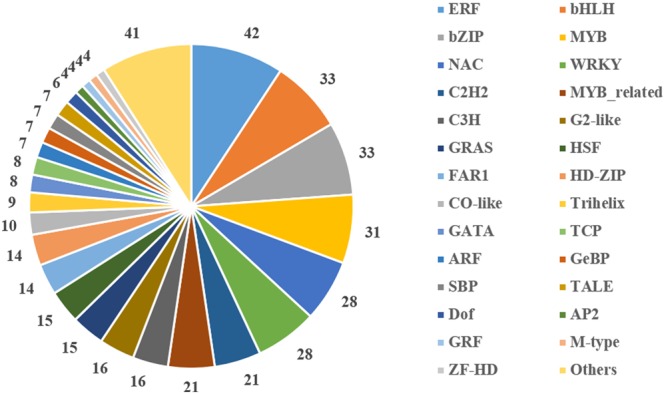
**Distribution of differentially expressed transcription factors in gene families**.

**FIGURE 6:**
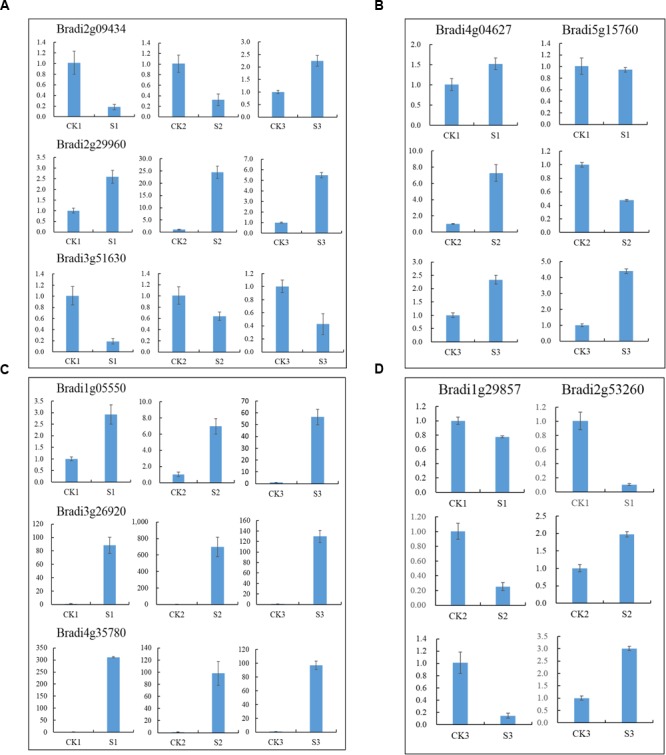
**Relative expression of selected TF genes. (A)** *ERF* genes, **(B)** *MYB* genes, **(C)** *HSF* genes and **(D)** *NAC* genes.

Transcription factors are important regulators that participate in the response to biotic and abiotic stresses (Lindemose et al., 2013). In order to better understand the molecular mechanism regulating the response to heat stress in *B. distachyon*, 454 TFs were identified from DEGs according to the rules of family assignment illustrated in PlantTFDB (Guo et al., 2008). These TFs belong to 49 TF families, such as ERF (42), bHLH (33), bZIP (33), MYB (31), NAC (28), and WRKY (28) (**Figure 5**). Compared with previously results (You et al., 2015), many *NAC* genes in S1 and S2 samples showed consistent trends in expression. For example, the expression of *BdNAC006* (*Bradi1g17440*), *BdNAC034* (*Bradi2g24987*), *BdNAC042* (*Bradi2g53260*), and *BdNAC062* (*Bradi3g47627*) was up-regulated by heat stress both in 3-week-old heat-treated seedlings (You et al., 2015) and in our S1 and S2 samples (Supplementary Table S4). Similarly, the expression of *BdNAC009* (*Bradi1g29857*), *BdNAC016* (*Bradi1g50057*), *BdNAC025* (*Bradi1g76207*), *BdNAC033* (*Bradi2g24790*), and *BdNAC043* (*Bradi2g55780*) was down-regulated in both 3-week-old heat-treated seedlings (You et al., 2015) and S1 and S2 samples (Supplementary Table S6). These results suggest that these *NAC* genes are involved in heat stress response. We selected 10 interesting TFs including three ERFs (*Bradi2g09434, Bradi2g29960, Bradi3g51630*), three HSFs (*Bradi1g05550, Bradi3g26920, Bradi4g35780*), two MYBs (*Bradi4g04627, Bradi5g15760*), and two NACs *(Bradi1g29857, Bradi2g53260*) for further validation. Results showed that the expression level of these genes was consistent with the RNA-Seq data (**Figure 6**).

### Identification of Alternative Splicing Events

Alternative splicing (AS) is a widespread co- and post-transcriptional regulatory process in plants. It can produce more than one isoform of mRNA transcripts and proteins from one gene (Stamm et al., 2005; Labadorf et al., 2010). AS is involved in most plant processes and is particularly prevalent in plants exposed to environmental stresses (Macaulay et al., 2015; Mandadi and Scholthof, 2015). To investigate the influence of heat on AS, we analyzed the events of AS with a threshold of false discovery rates (FDR < 0.05). In total, 1,973 (604, 1,082, and 287 events in S1, S2, S3, respectively) AS events were identified distributed across 451 DEGs, including 131, 320, 110 in S1, S2, S3 sample groups, respectively (Supplementary Table S5). Cassettes A3SS, A5SS, and RI are the most predominant AS types under heat stress (Supplementary Figure S1A). Of the 451 DEGs, 18 generated isoforms were identified in the three groups of samples after heat treatment (Supplementary Figure S1B). These AS genes were highly enriched for RNA splicing and protein folding BPs in GO enrichment analysis, while the spliceosome was most highly enriched in the KEGG pathway (Supplementary Figures S1C,D).

**FIGURE 7:**
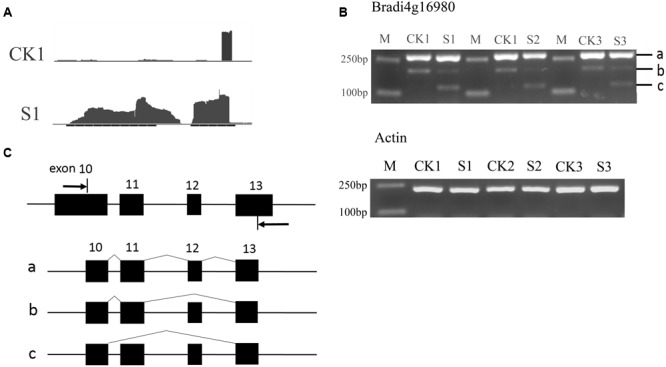
**Alternative splicing events in *Bradi4g16980*. (A)** The IGV map of RNA-seq reads. The reads showed that the middle exon was differentially spliced. **(B)** Result of semi-quantitative RT-PCR. **(C)** Diagram of isoforms a, b, and c. Arrows in **(C)** indicate primers.

**FIGURE 8:**
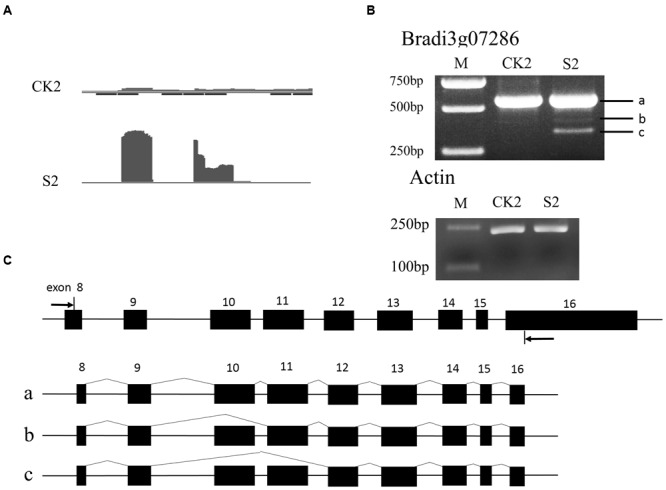
**Alternative splicing events in *Bradi3g07286*. (A)** The IGV map of RNA-seq reds of CK2 and S2, respectively. **(B)** Result of semi-quantitative RT-PCR of CK2 and S2. **(C)** Diagram of three alternative splicing isoforms. Arrows in **(C)** indicate primers.

To verify the AS events, the two genes *Bradi4g16980* and *Bradi3g07286* were selected for further analysis. The AS of *Bradi4g16980* can occur in a skipped exon manner, indicated by the Integrative Genomics Viewer (IGV) map of RNASeq reads (**Figure 7A**). According to the predicted sites and the length of exons, primers were designed and semi-quantitative RT-PCR experiments were performed. As shown in **Figures 7B,C**, in addition to bands *a* that included exons 10, 11, 12, and 13, and *b* that included exons 10, 11, and 13 that could be amplified in leaves of seedlings under normal conditions, band *c*, which included exons 10 and 13, was amplified in heat-treated samples. This result indicated another isoform was formed during the RNA processing by skipping the 12th exon. Similarly, in *Bradi3g07286*, the data show the occurrence of AS (**Figure 8A**). The PCR products from leaves at the heading stage only contained one band; however, after heat treatment, another two bands emerged (**Figure 8B**), indicating that heat stress induced AS. Analysis of the genome sequence suggested that band *b* product excluded the 10th exon that was included in band *a* product; while band *c* excluded both 10th and 11th exons (**Figure 8C**).

## Discussion

### The Influence of Heat Stress on Plants Is Complicated

High temperature and drought are perhaps the two most serious environmental factors that limit crop growth and yield worldwide, and the combination of these stresses can cause many physiological changes that affect crop yield and quality (Rizhsky et al., 2002, 2004; Mittler, 2006; Prasad et al., 2011; Vile et al., 2012), especially at the reproductive stage. In barley, heat stress significantly increases the number of aborted spikes and decreases kernel weight (Rollins et al., 2013). Heat stress causes a significant decrease in grain number, spikelet fertility, grain yield and harvest index as well as chlorophyll content (Prasad et al., 2011). In maize and spring wheat, heat stress decreases pollen fertility and grain number (Westgate, 1994; Prasad et al., 2011). Additionally, heat stress affects a series of physiological traits in many plants. For example, it inhibits photosynthesis mainly via altering non-stomatal traits, such as electron transport capacity and Rubisco activity (Salvucci and Crafts-Brandner, 2004a,b; Way and Oren, 2010). In cotton, the photosynthetic rate and stomatal conductance decreases and leaf temperatures increase under heat stress (Carmo-Silva et al., 2012). These observations demonstrate the complicated influence of high temperature on plants.

At the molecular level, the expression levels of more than one-fourth of the genes in the *B. distachyon* genome (6,609/25,532) were altered after heat treatment (**Figure 1**). These genes are involved in cell membranes, chloroplasts, the endoplasmic reticulum, and other organelles. Proteins encoded by these DEGs play multiple roles and participate in constituting spliceosomes, photosystem I, photosystem II, and so on. In addition to involvement in the response to heat and other abiotic stresses like hydrogen peroxide and chitin, the result of GO enrichment analysis showed that these DEGs were involved in 12 other BPs such as photosynthesis, protein dephosphorylation, and protein folding (**Figure 4**). The processes of photosynthesis, protein processing, and mRNA splicing were especially present in this gene analysis. GO enrichment analysis indicated that in response to heat; most of the up-regulated genes were involved in protein folding, protein processing, the spliceosome, and some physiological metabolic pathways. The GO terms related to photosynthesis were enriched in genes that were down-regulated in response to high temperature. Of the 6,609 DEGs, 454 genes encoded different types of TFs that regulate plant development and growth, and the response to biotic and abiotic stresses by activating or repressing the expression of their target genes. These results are similar to what has been shown in rice and bread wheat, where the expression levels of a majority of genes are changed under heat stress.

Additionally, some reports show that the expression profiles of miRNAs involved in plant growth and development are altered under high temperature (Xin et al., 2010; Kumar et al., 2015b). The altered expression of miRNAs has been implicated in the effects on plant growth and development during environmental stress (Ding et al., 2013). Recently, Kumar et al. (2015b) proposed that the expression of heat-responsive miRNAs was induced by elevated temperature, which in turn repressed the expression of their target genes and TFs. Taken together, previous work and our study suggest that the heat stress affects many BPs and pathways by altering the expression of genes that encode TFs and miRNAs.

### Heat Stress Induces Alternative Splicing

Alternative splice sites are used to generate two or more mRNA product from one pre-mRNA transcript (Staiger and Brown, 2013) to improve the diversity of functional proteins (Sablok et al., 2011). High throughput sequencing for transcript profiling in plants has revealed that AS affects a much higher proportion of the transcriptome than originally predicted. RNA-Seq technologies have greatly facilitated the comprehensive survey of AS events (Sablok et al., 2011; Walters et al., 2013). AS is very popular in plants and the frequency of AS in Arabidopsis for example, is more than 61% of intron-containing genes under normal growth conditions (Marquez et al., 2012); and in *B. distachyon*, AS is also common (Sablok et al., 2011). Walters et al. (2013) identified 1,219 AS events from 941 genes and Dou et al. (2015) identified AS events in two MADS-box genes. Some studies indicate that temperature can regulate AS and modulate the activity of splicing regulators and induce the AS of some genes in plants (Kwon et al., 2014; Capovilla et al., 2015). For example, at 31°C, one isoform of the *Arabidopsis* gene *HsfA2, HsfA2 II*, was generated by splicing a 31-bp exon within one conserved intron. When the temperature rose to 42°C, another isoform, *HsfA2 III*, was formed (Sugio et al., 2009). In rice, high temperature also induces AS. A temperature and drought-responsive gene, *DREB2B* generates a functional protein by AS. Under normal conditions, inclusion of the 53-bp exon 2 introduces a frame shift and premature termination, which leads to a non-functional transcript isoform (*OsDREB2B1*). Upon exposure to high temperatures, exon 2 is skipped and an ORF (*Os-DREB2B2*) only including exon 1 and 3 is formed (Matsukura et al., 2010).

In the present study, 46 DEGs associated with the spliceosome were identified in total. Of them, the expression of 43 DEGs was up-regulated under heat stress and 16 DEGs including three genes encoding splicing factor proteins were identified in our three sample groups. In particular, the expression level of one gene encoding one splicing factor in the Prp18 protein family was up-regulated 114.68611, 9,389.8651, and 1,894.9526 times in the three treatment groups compared with the control. These results suggest that the ability for RNA splicing is enhanced at high temperatures. Likewise, 1,973 AS events distributed between 451 DEGs were identified (Supplementary Table S5) after heat treatment. This result clearly shows that high temperature induces AS by up-regulating the expression of genes involved in RNA splicing. In fact, plants can adjust the abundance of functional proteins through AS. Most likely some proteins are not necessary under normal conditions, but when the environmental conditions change, plants need some proteins to respond to the new conditions. In this case, the expression of genes relative to RNA processing and splicing are up-regulated and more AS events occur. Thus, heat stress not only regulates the expression of genes at the transcriptional level, but also at the post-transcriptional level. Of course in addition to AS, other manners of post-transcriptional regulation could include alternative protein folding and modification of proteins.

### The Molecular Mechanism in Response to Heat Stress Is Conserved

In order to identify the responsive mechanism and commonalities in response to heat stress among different monocots, we compared our data with previous studies in rice (Zhang et al., 2012, 2013), wheat (Qin et al., 2008; Liu et al., 2015), maize (Fernandes et al., 2008) and switch grass (Li et al., 2013). In these species, protein folding, responses to abiotic stimulus, cellular and primary metabolic processes were significantly enriched BP identified in the associated GO terms. Furthermore, MF in binding associated chaperone and catalytic activity were enriched in all of the monocots tested, including *B. distachyon*.

In total, 885 and 395 homologous heat responsive genes from rice flag leaves and panicles were found in S2 and S3 samples of *B. distachyon* DEGs in this research, respectively.

To accommodate the imbalance and to survive in the extreme environments, plants need to reestablish their transcriptome, proteome, and metabolites (McClung and Davis, 2010; Mittler et al., 2012; Li et al., 2013). HSPs and other chaperones play a key role in protein–protein interactions such as folding, assisting in proper protein conformation, stabilizing partially unfolded proteins, and prevention of unwanted protein aggregation (Kotak et al., 2007; Li et al., 2013). To judge the conservation of molecular mechanisms in response to heat stress further, a comparison between our data (S2 and S3) and previous rice data (Zhang et al., 2012, 2013) was conducted. There were at least 83 genes encoding HSPs and other chaperones that were strongly induced by heat stress in *B. distachyon* (Supplementary Table S6), which is consistent with results that show that about 50% of homologous rice genes were also observed in heat-treated flag leaves or panicles (Supplementary Table S6).

The expression of many TF genes was altered by heat stress. In *B. distachyon*, 454 differentially expressed TFs were distributed in 49 families were identified. There were 13 families that had more than 15 genes in each; these families included *ERF* (9.25%), *bHLH* (7.27%), *bZIP* (7.27%), *MYB* (6.83%), *NAC* (6.17%), and *WRKY* (6.17%). Similarly, most of these TF families were the most significantly responsive TF genes observed in rice (Zhang et al., 2012, 2013) and wheat (Qin et al., 2008).

## Author Contributions

Experimental design: HL; Experiments: SC and HL; Date analysis: HL and SC; Manuscript preparation: HL and SC; Supervision, fundings and reagents: HL.

## Conflict of Interest Statement

The authors declare that the research was conducted in the absence of any commercial or financial relationships that could be construed as a potential conflict of interest.

## Footnotes

1 http://www.bioinformatics.babraham.ac.uk/projects/fastqc

2 http://www.ncbi.nlm.nih.gov

3 http://www.uniprot.org/

4 http://www.geneontology.org/

5 http://www.kegg.jp/

6 http://planttfdb.cbi.pku.edu.cn/download.php
