# Supplementary material for: Heat Stress Regulates the Expression of Genes at Transcriptional and Post-Transcriptional Levels, Revealed by RNA-seq in Brachypodium distachyon
Source: Front Plant Sci. 2017 Jan 10;7:2067. doi: 10.3389/fpls.2016.02067 (PMC5222869; doi:10.3389/fpls.2016.02067)
Supplement: Supplementary file 2 [file Image_1.pdf]

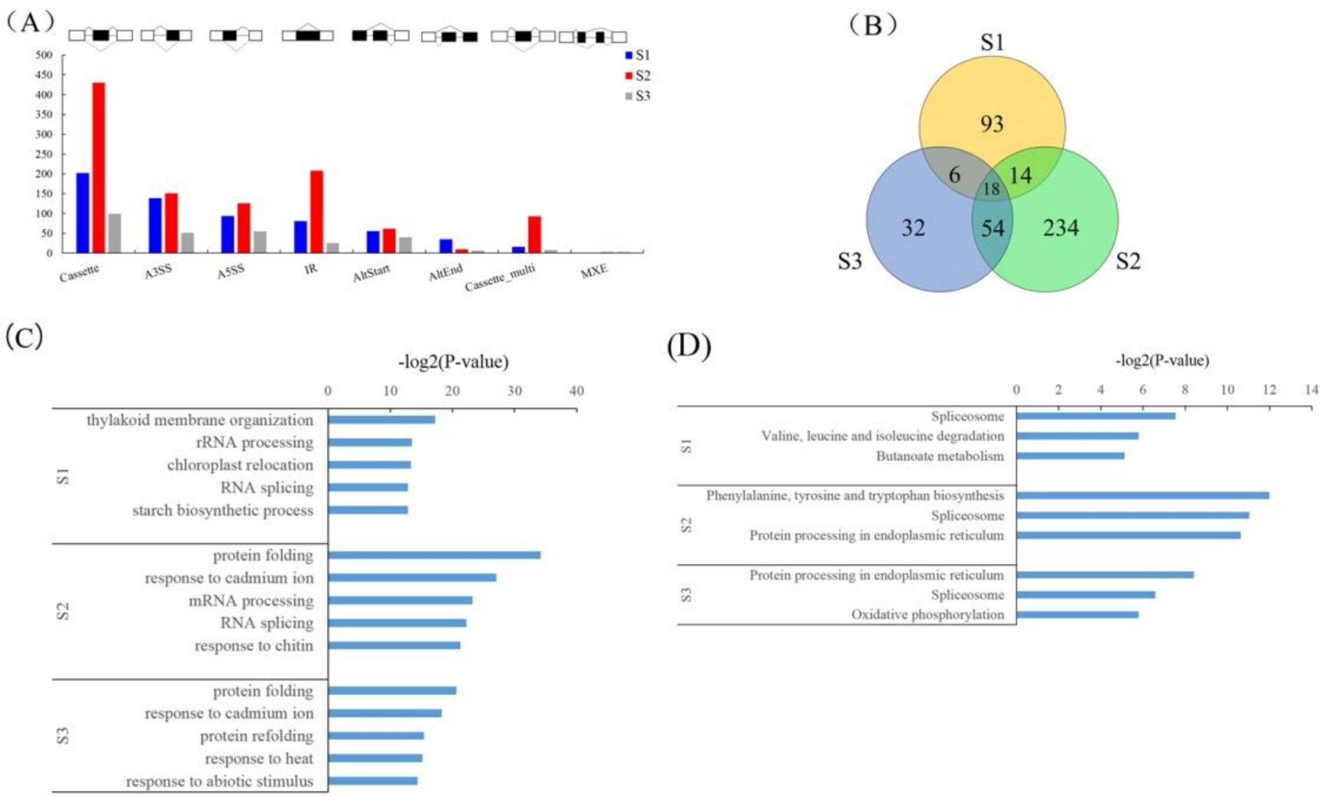

Figure S1. The alternative splicing events from RNA-seq data and function enrichment analysis of AS genes

(A) Statistics of expressed alternative splicing events in S1, S2, and S3. (B) AS events distributed in DEGs among S1, S2, and S3. (C) GO enrichment analysis for the AS genes. Only "Biological process" category was listed. (D) KEGG pathway analysis for the AS genes.
